# The Blood Concentration of Metallic Nanoparticles Is Related to Cognitive Performance in People with Multiple Sclerosis: An Exploratory Analysis

**DOI:** 10.3390/biomedicines11071819

**Published:** 2023-06-25

**Authors:** Marcela de Oliveira, Felipe Balistieri Santinelli, Paulo Noronha Lisboa-Filho, Fabio Augusto Barbieri

**Affiliations:** 1 Medicine and Nanotechnology Applied Physics Group (GFAMN), Department of Physics and Meteorology, School of Sciences, São Paulo University (Unesp), Bauru 17033-360, SP, Brazil; paulo.lisboa@unesp.br; 2 REVAL Rehabilitation Research Center, Faculty of Rehabilitation Sciences, Hasselt University, 3500 Hasselt, Belgium; felipe.balistierisantinelli@uhasselt.be; 3 Human Movement Research Laboratory (MOVI-LAB), Department of Physical Education, School of Sciences, São Paulo State University (Unesp), Bauru 17033-360, SP, Brazil; fabio.barbieri@unesp.br

**Keywords:** cognition, multiple sclerosis, processing speed of information, attention, memory, nanoparticles, metal, neuropsychological tests, biomarkers, blood

## Abstract

The imbalance in the concentration of metallic nanoparticles has been demonstrated to play an important role in multiple sclerosis (MS), which may impact cognition. Biomarkers are needed to provide insights into the pathogenesis and diagnosis of MS. They can be used to gain a better understanding of cognitive decline in people with MS (pwMS). In this study, we investigated the relationship between the blood concentration of metallic nanoparticles (blood nanoparticles) and cognitive performance in pwMS. First, four mL blood samples, clinical characteristics, and cognitive performance were obtained from 21 pwMS. All participants had relapse–remitting MS, with a score of ≤4.5 points in the expanded disability status scale. They were relapse-free in the three previous months from the day of collection and had no orthopedic, muscular, cardiac, and cerebellar diseases. We quantified the following metallic nanoparticles: aluminum, chromium, copper, iron, magnesium, nickel, zinc, and total concentration. Cognitive performance was measured by mini-mental state examination (MMSE) and the symbol digit modalities test (SDMT). Pearson’s and Spearman’s correlation coefficients and stepwise linear regression were calculated to assess the relationship between cognitive performance and blood nanoparticles. We found that better performance in SDMT and MMSE was related to higher total blood nanoparticles (r = 0.40; *p* < 0.05). Also, better performance in cognitive processing speed and attention (SDMT) and mental state (MMSE) were related to higher blood iron (r = 0.44; *p* < 0.03) and zinc concentrations (r = 0.41; *p* < 0.05), respectively. The other metallic nanoparticles (aluminum, chromium, copper, magnesium, and nickel) did not show a significant relationship with the cognitive parameters (*p* > 0.05). Linear regression estimated a significant association between blood iron concentration and SDMT performance. In conclusion, blood nanoparticles are related to cognitive performance in pwMS. Our findings suggest that the blood concentration of metallic nanoparticles, particularly the iron concentration, is a promising biomarker for monitoring cognitive impairment in pwMS.

## 1. Introduction

Multiple sclerosis (MS) is characterized by a demyelination process that occurs within the central nervous system (CNS), resulting in brain damage and atrophy [1]. Although MS is commonly associated with sensory and motor symptoms [2], cognitive impairment is a frequent manifestation already found in the early stages of the disease [3]. Cognitive impairment is a significant contributor to work-related problems and job loss and indicates a higher risk of future disability worsening [3,4]. The prevalence of cognitive impairment in people with MS (pwMS) ranges from 34% to 65% [5]. Cognitive impairment can be found across all MS phenotypes: 20–25% of individuals with a clinically isolated syndrome, 30–45% of individuals with relapsing–remitting MS, and 50–75% of individuals with secondary progressive MS display cognitive deficits [5,6]. The main cognitive domains affected by MS include cognitive processing speed, visual and verbal memory, executive function, and visuospatial processing [7]. However, changes in cognitive performance in MS are often overlooked, leading to delays in treatment adaptations and hindering the monitoring of disease progression. 

Understanding the causes of cognitive deficits and precisely determining cognitive impairment are critical for the effective treatment and management of MS. In a recent study by Rademacher et al. [8], an overview was provided on the use of biological markers to investigate their association with performance on cognitive tests in pwMS. Previous studies have suggested that inflammatory activity and neuro-axonal loss may play a role in cognitive function in MS [2]. Also, cognitive deficits such as defective cognitive processing speeds and impaired learning and memory have been linked to regional grey matter atrophy, the disruption of neural networks, and a lack of compensatory mechanisms in MS, as demonstrated by functional magnetic resonance imaging (MRI) [3]. High levels of iron deposition in the pulvinar, putamen, caudate nucleus, and globus pallidus have also been correlated with reduced cognitive performance in pwMS [9]. While these findings provide valuable insights to explain cognitive impairment in MS, brain imaging analysis protocols (e.g., MRI) can be expensive, inaccessible to all, and require complex techniques. Thus, the development of more affordable and accessible methods for diagnosis and assessment may aid in the early detection and treatment of cognitive impairment in MS. 

Diagnosing cognitive impairment in individuals with MS can be challenging. For example, clinicians must consider various factors such as psychiatric comorbidities, medication side effects, and MS symptoms that may negatively impact cognitive performance when making a diagnosis [3]. The mini-mental state examination (MMSE) and the symbol digit modalities test (SDMT) are commonly used tests to screen for cognitive deficits in MS. Considering that information processing speed and attention may be impaired early in MS [10], the SDMT seems to be the most effective single tool to assess cognition even in the initial stages of the disease [3,7]. The SDMT is widely recommended due to its sensitivity, reliability, and predictive validity in pwMS [3,7]. However, it should be noted that while the SDMT assesses information processing speed and attention effectively, it is not specific enough to evaluate other cognitive domains such as working memory, short- and long-term memory, paired-associate learning, and visual scanning, which are also important for overall cognitive performance. 

Another commonly used tool, the MMSE, can provide screening of mental state, including memory and executive function. However, it is not sensitive or specific enough to comprehensively evaluate cognition in MS [10]. While simple neuropsychological tests are helpful for routine care for pwMS, they are not sufficient on their own to fully evaluate cognition in MS and do not present unified cut-off scores for cognitive impairment in MS [11] due to the involvement of multiple cognitive domains [10]. Therefore, the identification of biomarkers to complement the diagnosis of cognitive impairment is emergent in MS. Biomarkers can provide a more comprehensive and complete assessment of cognitive performance and aid in monitoring cognitive impairment over time. Integration of biomarkers into the diagnostic process has the potential to enhance the understanding and management of cognitive impairment in MS.

The role of an imbalance in the concentration of metallic nanoparticles in MS degeneration has been established [12]. Metallic elements are known to be neuroactive and can affect various parts of the body, including the CNS [13]. An imbalance in the concentration of metallic nanoparticles may have adverse effects on cellular function, leading to oxidative stress, cell death, and brain damage [9]. Previous studies have reported that around 90% of neurodegenerative diseases are associated with exposure to potentially toxic elements, including metals found in manufacturing, contaminating ecosystems, arable soils, air, and food [14]. Oliveira et al. [12] demonstrated a decrease in the blood concentration of beryllium, copper, chromium, cobalt, nickel, magnesium, and iron, as well as an increase in lead concentrations in pwMS. The low levels of metallic elements in the body play a critical role in various metabolic processes, the development of the nervous system, and the myelination of nerve fibers [15]. In addition, these low blood values may suggest the accumulation of these elements in the CNS, including brain iron accumulation [16]. Therefore, considering that the concentration of metallic nanoparticles has been proposed as a potential cause of neurodegeneration in MS [12], it is plausible to hypothesize that the blood concentration of metallic nanoparticles could also be related to cognitive impairment in pwMS. However, the specific role of the blood concentration of metallic nanoparticles in explaining cognitive dysfunction remains unclear and has not been extensively explored yet. On the other hand, there is promising potential for the use of nanomaterials in the treatment and diagnosis of MS, although their applications are still in their infancy [17]. In this manuscript, we present an exploratory investigation into the relationship between the blood concentration of metallic nanoparticles and cognitive performance, specifically focusing on information processing speed, attention, memory, and executive function in pwMS.

## 2. Materials and Methods

### 2.1. Participants

Two power analyses (G*power©) were calculated to determine the sample size of the study. The first analysis indicated a minimum of 19 participants required for correlation (power of 80%, alpha of 0.05, and Cohen’s effect size for a large correlation of 0.60). The second analysis indicated a minimum of 13 participants required for linear regression (power of 80%, alpha of 0.05, Cohen’s effect size for a large correlation of 0.64, and 2 predictors) [18,19]. Thus, 21 individuals with MS between 18–42 years of age were enrolled in the present study. The inclusion criteria were as follows: (i) a score of ≤4.5 points on the expanded disability status scale (EDSS) [20], (ii) relapse-free in the three previous months from the day of evaluation, and (iii) the absence of orthopedic, muscular, cardiac, and cerebellar diseases. All participants exhibited the relapse–remitting type of MS in accordance with the revised McDonald criteria [21]. Additionally, participants with other diseases that could potentially interfere with the method analysis were excluded.

### 2.2. Study Protocol and Data Analysis

The evaluation assessment procedures were performed in a single visit and standardized in the morning. First, height and weight were measured, and a blood sample was collected. This was followed by clinical evaluation and cognitive tests. The procedures were approved by the School of Sciences ethics committee of the São Paulo State University (3 November 2018—identification code: CAAE #99191318000005398), and all participants signed an informed consent form before data collection. 

Four milliliters (mL) of whole blood samples were obtained from all subjects following standard procedures. The samples were collected in dry tubes without an anticoagulant factor for the quantification of blood metallic nanoparticles. Subsequently, all samples were freeze-dried and stored deep-frozen until use. The analyses were conducted in accordance with the recommendations outlined in Oliveira et al.’s study [12]. In brief, approximately 0.2 g (equivalent to ~1.2 mL) of freeze-dried whole blood was added to a mixture containing 3 mL of nitric acid and 2 mL of hydrogen peroxide. The samples were then placed in closed vessels and digested using microwave-assisted digestion techniques, following these steps: (i) 15 min of temperature ramp up to 180 °C and (ii) 15 min duration at 180 °C. This type of procedure has been found to be highly effective for digesting organic samples and biological fluids [22]. After, the digested samples were diluted using a 1.2/25 (*v*/*v*) ratio and filled up to 25 mL with deionized water. The diluted samples were then analyzed using inductively coupled plasma—optical emission spectrometry (ICP-OES). The ICP technique, known for its high sensibility, wide dynamic range, and multi-element capability, has been widely utilized for quantifying various elements in bodily fluids [23]. Its application ensures accurate measurement of metals in the blood samples of this study. The reference whole blood sample was digested under the same conditions as the study samples and blank samples. Hydrogen peroxide and nitric acid were considered as blanks in the analytical method. The concentration of metallic elements, including aluminum, chromium, copper, iron, magnesium, nickel, and zinc, was quantified using ICP-OES. Also, the total blood concentration of metallic nanoparticles for each participant was calculated by summing the blood concentration of each metallic nanoparticle.

The EDSS [20], provided by the participants’ neurologists, was used for quantifying disability. This scale classifies individuals on a scale of 1 to 10. Scores from 1.0 to 4.5 indicate pwMS who are capable of walking without assistance, while scores from 5.0 to 10 represent individuals with walking impairment. Additionally, information regarding the time since the last relapse (relapse time) and MS onset (disease duration from onset) was also obtained.

The SDMT [24] and MMSE [25] were measured by trained staff to assess the cognitive level of the participants. The SDMT measures cognitive processing speed and attention. It involves a substitution task using a coding key with nine different abstract symbols, each paired with a numeral. A series of these symbols is presented below the key, and the participants are required to respond verbally with the corresponding number for each symbol. Prior to the actual test, the participants completed a practice session consisting of 10 items. During the practice session, any errors made by the participants were corrected by the evaluator, who explained the substitution of the symbol with its corresponding numeral based on the key. The actual test comprises completing as many as 110 items with a time limit of 90 s. The number of correct substitutions/responses made within this timeframe was recorded as the individual’s score. The SDMT has been previously validated for use in MS [7]. MMSE measures mental state and evaluates various aspects of cognitive status, including executive function, attention, language, memory, orientation, and visuospatial proficiency. It consists of an 11-question assessment that measures cognitive function across five areas: orientation, registration, attention and calculation, recall, and language. The maximum score achievable for MMSE is 30. MMSE has become widely adopted as a short screening tool for providing cognitive impairment in clinical, research, and community settings [26].

### 2.3. Statistical Analysis

Statistical analysis was conducted using IBM SPSS software version 26 (IBM Corporation, Armory, NY, USA), with a significance level set at *p* < 0.05. The normality of the cognitive data was assessed using the Shapiro–Wilk test. The SDMT exhibited a normal distribution (*p* = 0.201), while MMSE showed a non-normal distribution (*p* = 0.01). For the SDMT, Pearson’s correlation coefficient was applied to examine the relationship between cognitive performance and the blood concentration of metallic nanoparticles. For MMSE, Spearman’s correlation coefficient was used. The strength of the correlation was classified as weak (0 < r < 0.3), moderate (0.3 ≤ r < 0.5), or strong (r ≥ 0.5) [27]. Blood concentrations of metallic nanoparticles that exhibited significant correlation with cognitive variables were included in a stepwise linear regression model.

## 3. Results

The individual, clinical, and cognition characteristics and blood concentration of metallic nanoparticles are presented in Table 1. Additionally, individual data for the participants’ characteristics and blood concentration of metallic nanoparticles are shown in the Supplementary Material (Table S1).

**Figure 1:**
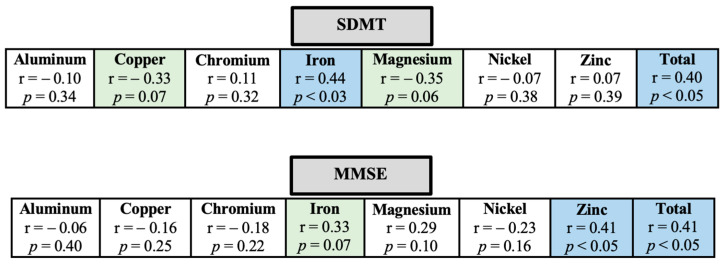
Relationship between cognitive performance (SDMT—symbol digit modalities test and MMSE—mini-mental state examination) and blood concentration of metallic nanoparticles in pwMS. The r and *p*-values are presented for each metallic nanoparticle. A blue square indicates a significant correlation while a green square indicates a tendency towards a significant correlation. Total—total blood concentration of metallic nanoparticles.

The SDMT and MMSE were related to the blood concentration of metallic nanoparticles (Figure 1). Specifically, better cognitive performance on the SDMT was moderately related to higher blood iron concentration and total blood concentration of metallic nanoparticles. In addition, magnesium and copper showed a relationship that approached the threshold of significance, indicating a trend towards a moderately negative relationship with the SDMT (*p* = 0.06 and 0.07, respectively). In the case of MMSE, better cognitive performance was moderately related to higher blood zinc concentration and total blood concentration of metallic nanoparticles. The relationship with iron concentration also approached significance (*p* = 0.07), suggesting a tendency towards a moderate association with MMSE. The other metallic nanoparticles, including aluminum, chromium, copper, magnesium, and nickel, did not show a significant association with the cognitive parameters in our study (*p* > 0.05).

**Figure 2:**
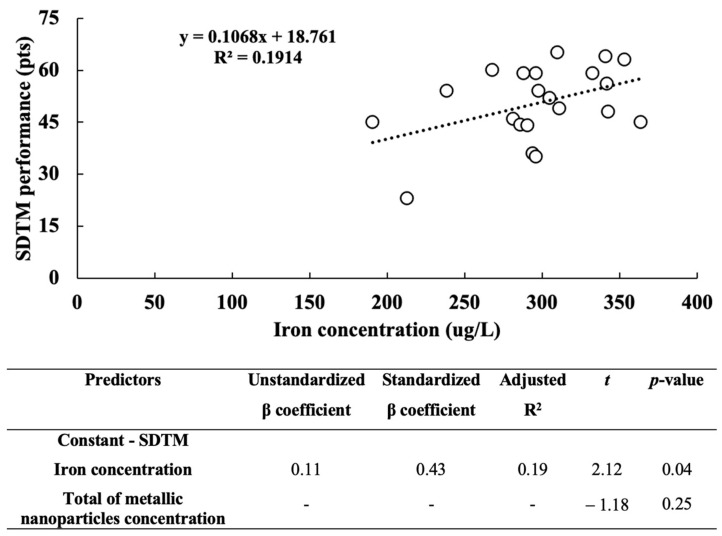
The relative contribution of blood iron concentration to the symbol digit modality test (SDMT) performance.

The linear regression analysis revealed a significant association between blood iron concentration and SDMT performance. The blood iron concentration explained 19.1% of the variance in the SDMT performance (Figure 2). No relationship was observed between MMSE performance and blood concentration of any metallic nanoparticles.

## 4. Discussion

Our study provided evidence supporting our hypothesis that a lower blood concentration of metallic nanoparticles is associated with reduced cognitive performance in pwMS. Specifically, we found that higher iron and zinc blood concentrations were moderately related to better cognitive performance on the SDMT and MMSE, respectively. Also, the blood iron concentration was a predictor of cognitive processing speed and attention performance (SDMT), explaining 19.1% of the variance in these cognitive abilities. These findings highlight the potential of the blood concentration of metallic nanoparticles as a biomarker for detecting cognitive impairment and complementing the diagnosis and monitoring of cognitive deficits in pwMS. 

There is a substantial gap between candidate/validated biomarkers and clinically useful biomarkers in MS, particularly concerning cognitive impairment. Having accurate and neurobiologically plausible biomarkers can greatly enhance diagnostics, predict disease outcomes, and facilitate the monitoring of MS progression [2]. During the cognitive decline preceding progressive MS, pwMS experience early or late subclinical cognitive decline. This decline is associated with a distinct set of biomarkers that can offer valuable insights into both diagnosis and treatment approaches [28]. Our findings may suggest that the blood concentration of metallic nanoparticles holds potential as a biomarker for identifying cognitive impairment in pwMS. Metals play essential roles in various biochemical processes and the normal functioning of the CNS [29]. Transition metals, such as copper, manganese, iron, and zinc, are known to be present in active protein sites serving as metabolic co-factors for structural and catalytic functions. They are also increasingly recognized for their involvement as second messengers in cell signaling [30]. In a previous study conducted by our group [12], we demonstrated decreased blood concentrations of beryllium, copper, chromium, cobalt, nickel, magnesium, and iron, along with an increased lead concentration in pwMS. In addition, the role of metals as risk factors in the etiopathogenesis of neurodegenerative diseases is now recognized, as well as their potential involvement to cause neuronal damage in MS [31]. Thus, it is plausible to propose that the imbalance in blood concentrations of metallic nanoparticles may serve as a biomarker in MS. 

Specifically for cognitive impairment, Brummer et al. [2] reported that higher levels of serum neurofilament light chain, which is a marker of neuro-axonal injury, were correlated with worse information processing speed, a key deficit underlying cognitive dysfunction in MS [3]. Serum neurofilament light chain levels have emerged as a fluid biomarker for neuro-axonal damage in MS and have been shown to predict disability progression [32]. Recently, a systematic review and meta-analysis identified several potential molecular biomarkers, such as neurofilament light chain and vitamin D, for cognitive performance in pwMS [8]. Our study adds to the understanding of the potential role of metallic nanoparticles in cognitive performance.

Metals play an indispensable role in reducing neuronal–axonal damage. For example, iron, copper, and zinc metals are crucial co-factors in the metalloproteins involved in myelin synthesis and neurotransmitter synthesis within the CNS [29,33]. In addition, the blood–brain barrier controls the transport of metal elements into the brain [34], which is regulated by the homeostasis of metals, especially zinc [35]. Specifically, metal ions interact with specific receptors in the endothelial lining, such as DMT1 and zinc transporters (ZIP8 and ZIP10), to cross the barrier and directly interact with cellular components in the CNS [31]. This interaction can lead to mitochondrial imbalance and increased production of reactive oxygen species, contributing to oxidative stress [31]. Disruption of metal homeostasis changes the permeability of the blood-brain barrier, allowing increased translocation of metals from the blood into the brain, which can exacerbate oxidative stress and other processes [36]. This imbalance in oxidative stress can trigger a pro-inflammatory response in microglia, characterized by the production of cytokines like IL-1, IL-6, and TNF-alpha, ultimately affecting neuronal viability and myelin production [31]. It is important to consider that the concentration of metals in the blood may indirectly reflect their concentration in the brain and be associated with neurodegeneration processes [37], including cognitive damage. For example, in our study, we observed a reduction in the blood concentration of metallic nanoparticles, which may suggest a higher brain concentration of these nanoparticles. Previous studies have linked higher brain concentrations of metallic nanoparticles to cognitive impairment [38]. Therefore, investigating the relationship between the blood concentration of metallic nanoparticles and the cognitive status of individuals with MS holds promise as a tool for monitoring cognitive progression in MS. 

Lesion burden has been identified as a predictor of cognitive disability in MS. Metal deposition is one of the factors contributing to brain lesions, which can have adverse effects on cellular function, including altering protein production and inducing cell death [39]. In addition, Metal ions such as zinc, iron, and copper have also been implicated in the aggregation of Aβ-amyloid and α-synuclein, triggering neurodegeneration [40]. Specifically, we showed that higher blood iron concentration was related to better attention and information processing speed (SDMT), while higher blood zinc concentration was related to better mental state (e.g., memory and executive function) (MMSE). Considering that the metal concentration in the blood and brain are interrelated, with higher levels in the blood corresponding to lower levels in the brain, and vice versa, our findings corroborated with Fujiwara et al.’s study [38], which showed a moderate association between increased iron levels in the globus pallidus and lower cognitive composite scores, including attention and information processing speed, in pwMS. However, it is worth mentioning that Modica et al. [9] did not find a relationship between iron concentration and attention and information processing speed in pwMS when accounting for regional atrophy. Thus, one may argue that the relationship between iron concentration and cognitive performance, particularly in attention and information processing speed, is still a topic of debate. Nonetheless, previous studies have shown that altered iron levels contribute to poor myelination, which is correlated with cognitive decline [41], underscoring the importance of iron concentration in cognitive impairment. 

Regarding zinc, the alterations in blood zinc concentration in pwMS are still conflicting: no alteration [42] vs. reduced concentration [15] compared to controls. Despite the inconsistencies in blood zinc concentration, it is important to note that alterations in zinc levels can have detrimental effects on neuronal cells, with both elevated and reduced levels being potentially neurotoxic and contributing to neurodegeneration [35]. Changes in blood zinc concentration may lead to increased extracellular zinc and decreased functioning of zinc in synaptic processes, which has been associated with cognitive decline [43]. Moreover, zinc distribution in the brain is region-specific, with certain areas such as the cerebral cortex and the hippocampus playing a role in cognitive processing [44]. It is worth mentioning that our study is the first to establish a relationship between blood zinc concentration and cognitive status in individuals with MS. However, further research is necessary to validate our findings and gain a better understanding of the potential effects of altered blood zinc concentration on cognition in pwMS. 

In short, our findings highlight the potential of blood concentration of metallic nanoparticles, particularly blood iron concentration, as a promising tool for monitoring cognitive impairment in pwMS. The analysis of metallic nanoparticle levels may be one of the ways to confirm cognitive impairment in people with MS, helping in both the diagnosis and monitoring of cognitive performance. In addition, our findings have significant implications for the lifestyle of pwMS. These findings emphasize the importance of reducing metal exposure from the environment, food, and industry to mitigate potential adverse effects. Thus, conducting longitudinal studies would provide valuable insights into the stability and progression of the relationship between metals and cognitive patterns over time in MS. Monitoring cognitive performance and metallic nanoparticle concentrations longitudinally would strengthen the robustness of our findings. Despite promising results, our study should be analyzed cautiously and considered preliminary. Although we had an effective sample size, the inclusion of a small sample of pwMS may impact the statistical power of our analysis. However, we observed moderate correlations and significant relationships (regression) even with a small sample, which is promising. Furthermore, future studies should expand on this analysis by including a broader range of metals, such as manganese, cobalt, and lead. Also, our findings should be validated with a larger sample size and across the full spectrum of MS disability levels, as our study focused on individuals with low-to-moderate disability (EDSS 1 to 4.5). In addition, our sample has no greater cognitive decline. Testing if our findings are consistent in pwMS with more pronounced cognitive decline is critical. 

## 5. Conclusions

In conclusion, our study demonstrated a significant relationship between the blood concentration of metallic nanoparticles and cognitive performance in pwMS. Specifically, we found that a higher blood iron concentration is associated with better cognitive processing speed and attention, as measured by the SDMT. Also, blood zinc concentration is related to higher mental states, including memory and executive function, as measured by MMSE. These findings suggest that the blood concentration of metallic nanoparticles holds promise as a biomarker for cognitive impairment in pwMS.

## Figures and Tables

**Table 1:** Means, standard deviations, maximum and minimum values of individual, clinical, and cognition characteristics, and the study participants’ blood concentration of metallic nanoparticles.

Individual Characteristics	
Sex (female/male)	12/9
Age (years)	32 ± 7 (45–18)
Height (m)	1.68 ± 0.02 (1.89–1.55)
Body mass (kg)	74.4 ± 3.1(94.6–51.1)
Clinical characteristics	
EDSS (points)	2.5 ± 1.1 (4.5–1.0)
Relapse time (months)	38 ± 30 (94–3)
Multiple sclerosis onset (months)	92 ± 77 (276–5)
Cognitive characteristics	
SDMT (points)	50.49 ± 10.70 (65–23)
MMSE (points)	29.05 ± 0.80 (30–28)
Blood concentration of metallic nanoparticles	
Aluminum (ug/L)	7.56 ± 2.52 (16.18–4.39)
Copper (ug/L)	0.98 ± 0.39 (1.78–0.44)
Chromium (ug/L)	0.39 ± 0.11 (0.65–0.29)
Iron (ug/L)	297.16 ± 43.84 (363.58–190.74)
Magnesium (ug/L)	28.69 ± 5.59 (41.77–20.03)
Nickel (ug/L)	0.31 ± 0.48 (1.69–0.04)
Zinc (ug/L)	3.20 ± 0.85 (4.58–1.63)
Total (ug/L)	338.30 ± 42.23 (404.92–235.56)

EDSS—expanded disability status scale; SDMT—symbol digit modalities test; MMSE—mini-mental state examination.

## Data Availability

The data presented in this study are available in this article.

## Supplementary Materials

The following supporting information can be downloaded at: https://www.mdpi.com/article/10.3390/biomedicines11071819/s1, Table S1: Values of individual, clinical, and cognition characteristics, and the study’s participants’ blood concentration of metallic nanoparticles.
