# Supplementary material for: The Blood Concentration of Metallic Nanoparticles Is Related to Cognitive Performance in People with Multiple Sclerosis: An Exploratory Analysis
Source: Biomedicines. 2023 Jun 25;11(7):1819. doi: 10.3390/biomedicines11071819 (PMC10376844; doi:10.3390/biomedicines11071819)
Supplement: Supplementary file 1 [file biomedicines-11-01819-s001.zip › biomedicines-2370297-supplementary.pdf]

Table S 1. Values of individual, clinical, and cognition characteristics, and the study's participants' blood concentration of metallic nanoparticles.

Al	Cu	Cr	Fe	Mg	Ni	Zn	Total	SDMT	MEEM	EDSS	Age	Onset	Relapse Time	Height	Mass	Sex
4.61	0.68	0.33	332.38	29.09	0.06	3.60	370.75	59	30	2.50	30	112	76	1.68	52.90	M
4.85	0.54	0.33	341.12	30.22	0.04	4.29	381.39	64	30	1.00	26	48	16	1.86	87.50	M
5.49	0.79	0.33	342.78	31.30	0.04	3.83	384.56	48	29	1.00	21	14	14	1.70	70.60	F
6.45	0.46	0.29	295.98	24.65	0.06	3.31	331.20	59	30	2.50	30	112	76	1.68	52.90	M
8.36	1.02	0.33	352.98	29.66	0.08	4.20	396.63	63	30	1.50	27	11	11	1.60	55.00	F
16.18	1.52	0.31	238.58	23.86	0.10	2.89	283.44	54	29	2.50	30	35	35	1.58	51.10	F
7.45	0.44	0.31	294.11	23.61	0.06	3.22	329.20	36	28	4.00	43	56	56	1.76	74.70	F
6.29	0.93	0.57	309.82	20.03	0.21	1.73	339.58	65	28	1.50	18	25	24	1.89	93.75	M
7.56	1.03	0.35	297.62	28.65	0.09	3.28	338.57	54	29	2.00	29	46	43	1.68	74.40	M
7.86	1.62	0.37	296.19	41.77	0.12	3.97	351.90	35	30	4.50	33	26	20	1.76	84.15	M
4.39	0.58	0.33	341.95	27.29	0.04	3.83	378.41	56	29	1.00	27	96	80	1.72	69.75	M
7.22	1.46	0.31	212.99	36.28	0.10	3.02	261.38	23	29	3.00	35	182	94	1.58	93.70	F
8.17	0.48	0.33	311.17	28.87	0.06	3.29	352.37	49	28	1.50	38	235	3	1.65	69.65	F
8.80	1.10	0.33	267.70	40.29	0.10	3.64	321.96	60	28	4.00	41	174	5	1.82	94.60	M
7.86	0.85	0.33	363.58	27.64	0.08	4.58	404.92	45	30	2.00	39	94	60	1.59	72.50	F
6.99	1.10	0.31	190.74	33.80	0.10	2.52	235.56	45	29	4.50	45	156	84	1.62	73.50	F
5.46	1.20	0.54	281.22	26.29	0.58	1.96	317.25	46	28	4.00	23	33	1	1.69	93.60	F
7.75	0.92	0.56	288.08	23.02	0.75	1.96	323.04	59	29	2.50	42	276	60	1.77	76.45	M
8.68	1.78	0.65	304.82	23.64	1.54	3.54	344.65	52	28	3.50	33	65	11	1.58	65.50	F
7.53	1.08	0.45	285.80	28.22	0.55	2.91	326.55	44	29	3.00	37	130	40	1.60	90.75	F
10.87	1.02	0.57	290.78	24.38	1.69	1.63	330.94	44	30	2.00	30	5	5	1.55	60.00	F

Note: *Concentrations of metallic elements (Al, Cu, Cr, Fe, Ni, Zn, and Total) in ug/L. SDMT, EDSS, and MMSE in points. Relapse time and onset in months. Age in years. Height in meters. Mass in Kilograms.*
